# Remission: does it already exist in chronic rhinosinusitis with nasal polyposis?

**DOI:** 10.1186/s40463-023-00657-2

**Published:** 2023-07-28

**Authors:** Yvonne Chan, Andrew V. Thamboo, Joseph K. Han, Martin Desrosiers

**Affiliations:** 1 Department of Otolaryngology-Head and Neck Surgery, Temerty Faculty of Medicine, St. Michael’s Hospital, University of Toronto, 30 Bond Street, Unit 8-163 CC North, Toronto, ON M5B 1W8 Canada; 2 Division of Otolaryngology-Head and Neck Surgery, Department of Surgery, University of British Columbia, Vancouver, BC Canada; 3 Department of Otolaryngology-Head and Neck Surgery, Eastern Virginia Medical School, Norfolk, VA USA; 4 Division of Otolaryngology-Head and Neck Surgery, Centre Hospitalier de L’University de Montreal, Montreal, QC Canada

**Keywords:** Chronic rhinosinusitis, Nasal polyposis, Endoscopic sinus surgery, Remission, Inflammatory bowel diseases, Asthma, Epithelium, Type 2 inflammation

## Abstract

**Background:**

Remission, defined as absence of symptoms and objective markers of disease, is emerging as the penultimate goal in the management of several chronic diseases. The concept of remission, well-established in Rheumatology as well as Gastroenterology, is currently emerging in Respiratory Medicine for asthma. It is interesting to consider whether the disease remission concept might successfully be applied to Otolaryngology-Head and Neck Surgery in the management of chronic rhinosinusitis with nasal polyposis (CRSwNP).

**Objective:**

The purpose of this letter is to explore the evidence supporting the concept of remission under continued medical therapy in chronic rhinosinusitis with nasal polyposis.

**Methods:**

The authors reviewed the literature and summarized studies in chronic rhinosinusitis with nasal polyposis evaluating for evidence of clinical, biochemical, and endoscopic remission.

**Results:**

Findings of the studies revealed that endoscopic sinus surgery with continued medical therapy achieved remission in approximately 50% of all patients. CRSwNP patients after primary endoscopic sinus surgery were able to achieve remission in 72% of instances, however this drops to 42% for patients having revision sinus surgery. For CRSwNP patients with co-morbidities such as asthma and aspirin exacerbated respiratory disease, remission rate drops to 23% and 23.5%, respectively compared to non-asthmatic CRSwNP patients who present a remission rate under continued medical therapy of 60%.

**Conclusion:**

Remission of symptoms and evidence of disease under medical therapy is indeed a concept achievable in patients with CRSwNP, as demonstrated by studies in the literature. Various co-morbidities, notably asthma, apparently influence rate of remission. Better defining this outcome through consensus-based definitions will allow for the development of strategies in CRSwNP care that can help affected patients attain complete relief from clinical, biochemical, and endoscopic markers of CRS with judicious use of medication and surgery. Future efforts will attempt to improve on these outcomes by achieving symptomatic and endoscopic control of disease following cessation of therapy, potentially paving the way towards clinical remission or a ‘cure’ in CRS.

**To the editor,**

Remission, defined as complete control of symptomatic and objective markers of disease is an emerging goal of therapy in the management of several chronic conditions. Recent application of this concept to the management of inflammatory airway disease has promoted the concept of clinical remission, using a “treat to target” approach [1, 2]. It is interesting to consider whether the disease remission concept might successfully be applied in the management of chronic rhinosinusitis with nasal polyposis (CRSwNP).

The concept of remission, already well-established in Rheumatology and Gastroenterology (GI), is now emerging in Respiratory Medicine with the outcome of clinical remission under continued medical therapy a target in asthma [3]. In the treatment of asthma, success is increasingly defined as remission under therapy, with the elimination of exacerbations, stabilization of symptoms, and the possibility of normalizing inflammatory markers which indirectly reflect lung function and inflammation. Guidelines for inflammatory digestive diseases are similar to those in asthma, in terms of their symptomatic endpoints and rigorous control of disease [4]. However, for inflammatory bowel disorders, an additional endoscopic criterion which documents epithelial and mucosal recovery from disease is also included. This framework might offer a better characterization for the control criteria in CRSwNP incorporating symptom control signifying clinical remission, inflammatory markers normalization indicating biochemical remission, and endoscopic remission demonstrating normal sinonasal mucosa.

A consensus statement from tertiary Canadian rhinologists has previously combined symptomatic and endoscopic assessments to define success after ESS, with ‘optimal’ results reported as absence of symptoms and normal appearance of the sinus mucosa on sinonasal endoscopy [5] with continued medical therapy. However, it was unclear how often, or even whether, this ‘optimal’ outcome could be achieved. Two recent studies in CRS have used this clinical endpoint, which is very similar to the remission definition used in Gastroenterology for inflammatory bowel diseases. The first, which assessed outcomes after treatment of CRSwNP with endoscopic sinus surgery [6], and the second, evaluating refractory CRSwNP managed with long-term, low dose azithromycin [7], both used clinical endpoints very similar to remission definitions employed by GI: symptom control, evidence of mucosal healing, and even a histologic demonstration of resolution of disease using expression markers. After ESS, clinical endpoints resembling remission (no symptom more than 1/3 on a three-point grading scale and sinus endoscopy showing no more than minimal edema or secretions and no nasal polyposis) were attained in 50% of all subjects, but with different rates of remission according to clinical criteria. At four months after surgery, individuals undergoing primary ESS for CRSwNP attained remission in 72%, while those with a history of previous surgery showed lesser response, with 42% remission rate. Asthmatic subjects did considerably worse than non-asthmatic subjects: non-asthmatics attained remission in 60%, while patients with asthma or with aspirin exacerbated respiratory disease (AERD) only showed emission in 23% and 23.5% of cases, respectively. For the azithromycin trial, there was a 54% remission rate overall. Again, asthma was associated with a worse outcome. The remission rates were 88% for non-asthmatics, 38% for asthmatics, and only 14% for AERD patients. Individuals demonstrating a successful response were characterized by parameters of epithelial recovery and healing, approaching those of optimal control as suggested for inflammatory digestive diseases [8, 9].

These findings suggest that remission, defined as absence of symptoms and endoscopic markers of CRS is indeed a concept that can be currently attained in CRSwNP, even in patients who failed prior sinus surgery. Some patient groups apparently have more difficult evolution, and asthma emerges as an important comorbidity in CRSwNP patients. At the time of writing, there is not yet a definition for remission in CRSwNP. The authors postulate that complete remission is no symptom (clinical) or evidence of disease (objective) and no exacerbations while on any therapy including surgery, topical steroids, and biologics. Better defining this outcome through consensus-based definitions will allow for the identification of situations where patients have complete relief from their disease symptomatically in addition to biochemical and endoscopic normalization, essentially achieving clinical, biochemical, and endoscopic remission. In a long-term prospective study, 21.1% of the CRSwNP patients were found to have no recurrence of polyps after 1 sinus surgery [10].

This evolving concept promises to alter our assessment of therapies for CRS, and may modify our expectations in CRS prolonged remission allow us to eventually cease medication, leading to a long-term “cure” even in the absence of medical therapy? This would radically alter the cost–benefit equation of emerging therapies by allowing shorter courses of increasingly expensive medical therapies. Future research will aim to identify biochemical markers that will help stratify these patients in an objective manner in addition to their clinical symptoms and endoscopy findings.

## Data Availability

Not applicable.

## Abbreviations

AERD: Aspirin exacerbated respiratory disease
CRSwNP: Chronic rhinosinusitis with nasal polyposis
ESS: Endoscopic Sinus Surgery
GI: Gastroenterology
OHNS: Otolaryngology—Head & Neck Surgery
